# Pathophysiological Mechanisms of Asthma

**DOI:** 10.3389/fped.2019.00068

**Published:** 2019-03-19

**Authors:** Andrew Bush

**Affiliations:** Departments of Paediatrics and Paediatric Respiratory Medicine, Royal Brompton Harefield NHS Foundation Trust and Imperial College, London, United Kingdom

**Keywords:** biomarker, transcriptomics, bronchial biopsy, bronchial brushings, induced sputum, airway inflammation, asthma phenotype, endotype

## Abstract

The recent *Lancet* commission has highlighted that “asthma” should be used to describe a clinical syndrome of wheeze, breathlessness, chest tightness, and sometimes cough. The next step is to deconstruct the airway into components of fixed and variable airflow obstruction, inflammation, infection and altered cough reflex, setting the airway disease in the context of extra-pulmonary co-morbidities and social and environmental factors. The emphasis is always on delineating treatable traits, including variable airflow obstruction caused by airway smooth muscle constriction (treated with short- and long-acting β-2 agonists), eosinophilic airway inflammation (treated with inhaled corticosteroids) and chronic bacterial infection (treated with antibiotics with benefit if it is driving the disease). It is also important not to over-treat the untreatable, such as fixed airflow obstruction. These can all be determined using simple, non-invasive tests such as spirometry before and after acute administration of a bronchodilator (reversible airflow obstruction); peripheral blood eosinophil count, induced sputum, exhaled nitric oxide (airway eosinophilia); and sputum or cough swab culture (bacterial infection). Additionally, the pathophysiology of risk domains must be considered: these are risk of an asthma attack, risk of poor airway growth, and in pre-school children, risk of progression to eosinophilic school age asthma. Phenotyping the airway will allow more precise diagnosis and targeted treatment, but it is important to move to endotypes, especially in the era of increasing numbers of biologicals. Advances in -omics technology allow delineation of pathways, which will be particularly important in TH2 low eosinophilic asthma, and also pauci-inflammatory disease. It is very important to appreciate the difficulties of cluster analysis; a patient may have eosinophilic airway disease because of a steroid resistant endotype, because of non-adherence to basic treatment, and a surge in environmental allergen burden. Sophisticated –omics approaches will be reviewed in this manuscript, but currently they are not being used in clinical practice. However, even while they are being evaluated, management of the asthmas can and should be improved by considering the pathophysiologies of the different airway diseases lumped under that umbrella term, using simple, non-invasive tests which are readily available, and treating accordingly.

## Introduction: Approaching Airways Disease

The recent Lancet *Asthma* Commission (1) was predicated on the assumption that the term “asthma” was no more a diagnosis than is “arthritis” or “anemia.” It is an umbrella term that should be used to describe a constellation of clinical symptoms, namely wheeze, breathlessness, chest tightness and cough, and should be followed by the question “what sort of asthma is this?” Dissecting out the individual asthmas is increasingly important as novel biologicals with different modes of action are increasingly being deployed. The ultimate aim is to discover endotypes of asthma, but currently we have not yet got to this point. The importance of endotyping is illustrated by the extraordinary achievements when the endotypes and the gene-class specific sub-endotypes of cystic fibrosis (CF) were first separated from the generality of conditions with chronic airflow infection and inflammation. The result has been the specific, molecular therapies (2–4), none of which would have come to the bedside if they had been tested on every child with a chronic wet cough. It is also important, but largely outside the scope of this chapter, to set airway disease in the context of extra-pulmonary co-morbidities such as obesity, and environmental and lifestyle factors, such as adverse environmental exposures and adherence (5).

The conventional view of at least school age and adult asthma is that the root cause is airway inflammation, which leads to airway hyper-responsiveness, and, secondary to repeated episodes of inflammation, airway remodeling. However, a critical review of the evidence shows that this view is untenable. There is only a weak correlation at baseline between eosinophilic inflammation and bronchial hyper-responsiveness (6, 7). The anti-IgE monoclonal omalizumab reduces airway eosinophilia, but has no effect on bronchial hyper-responsiveness (8), whereas the anti-TNF monoclonal etanercept reduces hyper-responsiveness but has no effect on airway inflammation (9). Furthermore, there is no relationship between the extent of airway remodeling, specifically reticular basement membrane thickness, and the degree or duration of any inflammatory parameter (10). Indeed, there is evidence that remodeling may be protective under some circumstances, discussed in more detail below. Thus, the relationships between the three classic components of asthma are more complex than previously thought, and this is highly relevant to considerations of pathophysiology.

## First Principle: Deconstructing Airway Disease

Adverse stimuli can affect any biological tube in relatively limited and stereotypical ways. These are:

- Narrowing to cause fixed obstruction
- Narrowing to cause variable obstruction which changes spontaneously over time, and with treatment
- Inflammation with various cell types predominant; inflammation may be harmful or beneficial
- The tube may become infected with combinations of bacterial, viral and fungal pathogens

- There may be increased “twitchiness” of the tube—this is different from variable obstruction. An increased reflex expulsive effort (cough) may not be accompanied by transient airflow obstruction
- The tube contents may be abnormal: including being too wet, too many solids, or too dry.

Furthermore, there are domains of risk, which also need to be considered in any discussion of pathophysiology:

- Risk of acute asthma attacks, which may be fatal
- Risk of impaired trajectories of lung growth, which may sometimes but not inevitably be associated with asthma attacks
- (in pre-school children) risk of progressing from episodic wheeze to eosinophilic atopic school age asthma
- A fourth risk, about which little is known and will not be discussed here, is the risk of failing to remit

Clearly not all are relevant to all pediatric airways diseases: the hallmark of CF is the effects of the airway being too dry [“low volume hypothesis” (11)] and infection and neutrophilic inflammation, whereas some at least of the asthmas are dominated by eosinophilic airway inflammation. What is also clear is that we need modern–omics or genetic tools to try to dissect out these components—and these are sadly lacking. Indeed, currently we are not even trying routinely to identify treatable traits in airway disease, instead haphazardly making diagnoses and embarking on therapeutic trials without making simple measurements in order objectively to phenotype the airway disease. The three important treatable traits, which will be considered in turn, are:

- Does the child have the treatable trait of eosinophilic airway inflammation which is likely to respond to inhaled corticosteroids (ICS)?
- Does the child have the treatable trait of (usually short-acting, β-2 agonist sensitive) reversible airflow obstruction? And conversely, does the child have the untreatable trait, meaning treatment should be discontinued, of fixed airflow obstruction?
- Does the child have the treatable trait of bacterial infection which is driving the disease and can be treated with antibiotics?

The contention of this chapter is that the isolated questions “does my child have asthma?” and (for example) “do survivors of preterm birth have a higher risk of asthma?” are meaningless in isolation. The correct questions are “does this child have an airway disease at all, or are the symptoms in fact due to deconditioning or some other cause (12)?” and, if the child has an airway disease, “what is the nature of this particular airway disease?”

## Current Best Practice: Phenotyping the Airway

A phenotype is defined as the set of observable characteristics of an individual resulting from the interaction of its genotype with the environment. It is important to make the distinction between phenotyping which is of clinical value (changes treatment, prognostic value) from those determining mechanistic pathways. However, if phenotyping does not help in either domain, it cannot be said to be useful. An endotype is defined as a subtype of a condition, which is defined by a distinct functional or pathobiological mechanism. In general, phenotyping leads to rather non-specific treatment, whereas endotyping opens up an exciting vista of pathway specific therapies, as I will show.

### Airway Inflammation, and the Potentially Treatable Trait of Airway Eosinophilia

#### Airway Eosinophilia

ICS are amongst the most effective agents in the whole of therapeutics for the vast majority of children with eosinophilic asthma. Low dose treatment, if taken efficiently and regularly, will result in complete control of asthma in most children (13), while accepting there are steroid resistant phenotypes. The most direct evidence of airway eosinophilia is of course obtained at fibreoptic bronchoscopy (FOB) with broncho-alveolar lavage (BAL) and endobronchial biopsy. This is of course not ethical or practical in most children, and non-invasive methods must be used. Induced sputum was initially the most popular technique, and although this is time-consuming, it is perfectly feasible as a diagnostic test for infection even in resource poor areas (14). Although in older children sputum and BAL eosinophilia are tightly correlated (15), this is not the case in pre-schoolers (16). Furthermore, there is a failure rate of up to 20% (17), and induced sputum does not reflect mucosal inflammation.

Of the other non-invasive methods, peripheral blood eosinophil count has become most popular. It reflects BAL eosinophilia (15, 18), and, even more importantly, is an excellent biomarker predicting response to anti-TH2 monoclonal antibody strategies (19–21). Peripheral blood eosinophil count can be measured on a finger prick sample with point of care equipment (22). Significantly, in the first attempt at personalized medicine in pre-school children, the combination of a peripheral blood eosinophil count >300/μλ and aeroallergen sensitization was the strongest predictor of response to ICS (23). Exhaled nitric oxide (FeNO) has also been used as a surrogate for airway eosinophilia. The utility of this method in reducing asthma attacks has been demonstrated (24), but although there is a relationship with induced sputum eosinophil count, it varies between individuals and is inconsistent in the same individual over time (25). Clearly FeNO and induced sputum are complimentary and useful, but exactly how they should be used in combination is unclear.

However, the presence of airway eosinophils should prompt critical thought. Firstly, airway eosinophilia, although often related to Type 2 inflammation, is not synonymous with that endotype, and this needs to be borne in mind when contemplating anti-TH2 monoclonal therapies. In our cohort of severe, therapy resistant asthmatics, those with steroid resistant airway eosinophilia had very little evidence of ongoing secretion of the signature TH2 cytokines interleukin (IL)-4, IL-5 and IL-13, in either induced sputum supernatant, BAL or immunohistochemistry of endobronchial biopsy (26). Also, the U-BIOPRED group, using sputum transcriptomics in adult asthmatics documented a group which included patients with moderate sputum eosinophilia, who instead of having the expected TH2 handprint, were characterized by genes of metabolic pathways, ubiquitination and mitochondrial function, as well as, in another study, an IL-6 modulated pathway (below).

Furthermore, if eosinophils are present in the airway, their role or otherwise in disease causation should be carefully considered. In adults at least, eosinophilic bronchitis is a cause of chronic cough, but with no evidence of reversible airflow obstruction; treatment is with ICS, but not β-2 agonists (27). In a challenging study, airway biopsies were compared in patients with active asthma, normal controls, and patients who by any criteria had outgrown their previously diagnosed asthma (28). The airway wall histology, in terms of eosinophilia and reticular basement membrane thickness was the same irrespective of whether the patient had active asthma or had “outgrown” the disease. This leads to the challenging question as to what is the “X-factor” that is needed to convert airway eosinophilia into airway disease? At the moment this remains completely unknown.

Having said all this, clearly if there is no airway eosinophilia, it seems to make little sense to prescribe an anti-eosinophil strategy such as ICS. Although it is true that corticosteroids have numerous genomic and non-genomic effects (29) which hypothetically could be beneficial in airway disease, this has never been shown, and there may be potentially adverse effects in at least some airway diseases, for example reducing neutrophil apoptosis and prolonging the survival of this cell in the airway (30). Surely in the twenty-first century we should not prescribe anti-eosinophilic medications if there is no airway eosinophilia to treat, any more than anti-hypertensives should be prescribed to people who have a normal blood pressure?

#### Neutrophilic Asthma?

This is another area which illustrates the danger of extrapolating adult studies to children. Asthma characterized by mucosal and sputum neutrophilia is well described in adults (31), who tend also to have severe asthma with less evidence of atopy. Unsurprisingly, neutrophilic asthma is steroid non-responsive. By contrast, in our cohort of children with severe asthma, multiple atopic sensitization was common, but there was no evidence of mucosal, sputum or BAL neutrophilia (26). However, in a subgroup of patients neutrophils were found within the epithelium (32), and, quite unlike what might be expected from adult data, these patient had better symptom control (Asthma control test, ACT) and better first second forced expired volume (FEV_1_) while being prescribed a lower dose of ICS. Although it is always dangerous to move from cross-sectional associations to hypotheses, nonetheless it would seem that, whatever the role of neutrophils in adult asthma, in pediatric asthma neutrophils are having a beneficial effect. This raises the intriguing possibility that bacterial infection may have a role in some pediatric asthmas. Again highly speculatively, is it possible that excessively high doses of ICS might actually worsen “bacterial asthma” (if it exists!) by causing topical mucosal immunosuppression (33), leading to a positive feedback loop of worsening symptoms leading to higher ICS doses leading to worsening symptoms? Further data are needed to explore this. However, a practical clinical message is that the finding of BAL or mucosal neutrophilia should prompt a search for another diagnosis.

### Fixed and Variable Airflow Obstruction

#### Variable Airflow Obstruction

Wheeze is a frequently sought, and often misinterpreted sound. Even when a polyphonic, musical predominantly expiratory noise is heard by the physician, all it betokens is narrowing of the airway lumen. It is not synonymous with airway smooth muscle constriction. Causes include intraluminal airway secretions; airway malacia which may be localized or generalized; and extraluminal compression, for example by a mass of lymph nodes. Most of the asthmas as defined above have the hallmark of either or both of obstructive physiology which improves with acute administration of a short acting β-2 agonist, or normal physiology but with an exaggerated response to stimuli such as cold air or allergen challenge.

In terms of variable airflow obstruction, as a profession we have been remiss in failing to document this objectively. Even pre-school children can perform spirometry, and criteria for adequate curves (34) and definitions of bronchodilator responsiveness (35) for this age group have been published. Simple field tests, like acute response to a short acting β-2 agonist, a short period of home peak flow or increasingly electronic spirometry monitoring, and an exercise challenge may be informative. Although there is no one definitive diagnostic test for asthma, and all the above have a high specificity but low sensitivity for asthma (36), the more tests that fail to demonstrated variable airflow obstruction, the less likely is the treatable trait of bronchodilator responsive airflow obstruction to be present.

AHR correlates poorly with inflammation (above), but of course there is a relationship which is best described by a two-component model, “inflammatory” and “anatomical.” Three major longitudinal studies (37–39) have measured AHR very early in life, before any significant exposure to infection or allergen, and certainly before there is any evidence of airway inflammation or remodeling (40). Each has shown a strong association between early AHR and adverse long term respiratory outcomes. The likely pathological basis is increased airway length and reduced radius, leading to a baseline increase in airway resistance (41), which with further narrowing by a constrictor stimulus leads to an exaggerated reduction in airflow. There is also evidence in later life that airway inflammation causes a component of AHR, and that anti-inflammatory therapies can improve AHR (42).

In terms of the practical value of measuring AHR in the clinic, population studies have shown that, although in group data, AHR relates to asthma severity, many normal people can be shown to have AHR but have no symptoms (43). Thus, “abnormal” AHR of itself does not lead to disease; by analogy with the airway eosinophil story, what is the “X-factor” that converts asymptomatic AHR to an airway disease? Again, this is completely unknown. However, what is certain is that failure to demonstrate AHR in a patient said to be symptomatic with asthma should lead to reconsideration of the diagnosis; certainly, if a child is thought to have eosinophilic airway inflammation but AHR cannot be demonstrated, then the child's symptoms are not due to eosinophilic asthma.

#### Fixed Airflow Obstruction

The importance of this *untreatable* trait is that ICS and other therapies should not be escalated when there is no hope of benefit. There is no generally accepted pediatric definition of fixed airflow obstruction. In general, it should be defined as an abnormal FEV_1_ (more than 1.96 Z-scores below normal) after a systemic corticosteroid trial and the acute administration of short-acting β-2 agonist, but neither the dose, duration or route of administration of systemic steroids, nor the dose of short-acting β-2 agonist is agreed. We use a single injection of intramuscular triamcinolone (40 mg in a child weighing <40 kgm, 80 mgm in the rest) to ensure adherence, and 1 mgm of salbutamol via a spacer (44). We found very little evidence of benefit from adding extra doses of triamcinolone (45). It should be noted that the measurement of steroid responsiveness in children with asthma encompasses more than just measuring spirometry; we use a multi-domain approach (46, 47).

One component of airway obstruction is determined antenatally. Maternal nicotine exposure (active and passive smoking, vaping) has been shown in animal models and humans to lead to structural changes with a readout of airflow obstruction shortly after birth, before the first viral infection (41, 48–52). Other adverse factors include maternal exposure to environmental pollution (53), maternal hypertension (54), and any factor leading to a low birth weight or prematurity or both (55, 56). The second component is the structural airway wall changes in established asthma, including increased airway smooth muscle, reticular basement membrane thickening, increased numbers of goblet cells and increased airway vascularity. Conventionally, these are considered to result from cycles of airway inflammation and contribute to airflow obstruction (above). However, at least in pediatrics, most studies linking inflammation and remodeling are cross-sectional and observational, and demonstrating association is a long step from proving causation. It is currently not possible to synthesis a coherent account of the pathophysiology of remodeling, and one can only present a few statements which require integration and explanation.

1. At least some aspects of remodeling, for example increased reticular basement membrane thickening, plateau in childhood, and are non-progressive into adult life (10).
2. No pediatric studies have shown eosinophilic airway inflammation with no evidence of remodeling. Remodeling has been described in the absence of current airway eosinophilia, but these children have been prescribed usually high dose ICS, and these data are equally consistent with the hypothesis of ICS having successfully treated airway eosinophilia as the alternative, that remodeling precedes airway eosinophilia (57).
3. Although airway remodeling can be partially attenuated by prolonged use of high-dose ICS (58, 59), the changes are much more steroid resistant than is airway eosinophilia.
4. Reticular basement thickening is inversely correlated with AHR, (60) and thus may be a protective response of the airway, protecting against life-threatening bronchoconstriction.
5. It is at least conceivable that reticular basement membrane thickening is a protective measure designed to limit penetration of cytokines and chemokines into the systemic circulation, and possibly also protect the airway mucosa from tissue damaging enzymes within the lumen (61).
6. On the other hand, the increase in airway smooth muscle in asthma (62), and the beneficial responses seen in adults with bronchial thermoplasty together with reduction in smooth muscle in animals submitted to thermoplasty (63, 64), suggests this aspect of remodeling is adverse.

### Airway Infection and the Asthmas

This is another area that is currently difficult to understand. Some issues are clear; acute attacks of wheeze are usually precipitated by respiratory viral infections at all ages (65). Recent studies have shown that bacteria are isolated equally frequently during a wheeze attack (66), but it is unclear whether bacteria cause the attack, or are the result of a transient mucosal immunoparesis secondary to viral infection or iatrogenic as a result of ICS treatment (33). The general failure of antibiotics to impact acute wheeze attacks to any great degree is a strong pointer against a causal role for bacteria. We also know that the airway microbiome differs between normal and asthmatic children (67), and that airway neutrophilia in children appears to be beneficial rather than the reverse (32), implying a more important role of infection than previously thought. It is also clear that very early nasopharyngeal bacterial colonization with bacteria is associated with a mixed TH1/TH2/TH17 mucosal response, and subsequent adverse respiratory outcomes (68–72), although whether bacterial colonization is causal, or a marker of a subtle immune deficiency, is unclear. In a study using bronchoscopy and BAL to measure airway inflammation and infection, and the airway microbiota, in severe pre-school wheezers, we demonstrated two separate microbiota-based clusters; a Moraxella positive, airway neutrophilic group, and a mixed microbiota, macrophage and lymphocyte predominant group (73). Interestingly, these did not relate to clinical phenotype or markers of atopic status. Long term follow up will be needed to determine the significance of these clusters. There are some *in vitro* data lending plausibility to a link between bacterial infection and airway eosinophilia; in a nasal polyp model, *Staph aureus* binds via TLR2 leading to epithelial release of the alarmins TSLP and IL33, and the TH2 signature cytokines IL5 and IL13 (74). However, we need more data about the role of bacteria and the interactions with airway eosinophilia to try to understand asthma pathophysiology.

Of more immediate practical clinical significance is the need to determine whether the child with respiratory symptoms has an underlying chronic bacterial infection which will respond to oral antibiotics [“persistent bacterial bronchitis (PBB)”] (75, 76). Typically the symptoms are of wet cough not wheeze, but secretions narrowing the lumen may cause wheeze, and non-wheeze noises may be misinterpreted by the family. For most of these children, invasive sampling is not appropriate. We have shown that the yield of organisms is much greater with induced sputum, even in young children, compared to cough swabs, and indeed induced sputum results are comparable to FOB (16). It should be noted that PBB, like bronchiectasis, is a description not a diagnosis, and should prompt a focused diagnostic work-up (77).

### The Time Domain: Often Unappreciated

The two important pathophysiological areas are firstly, the stability of measurements over time, and secondly, the extent to which sophisticated analysis of simple measurements over a long time period can help us understand asthma pathophysiology and understand risk.

#### Temporal Stability of Measurements

Adult studies in which sputum cell counts are used to guide treatment have given promising results, in particular in the reduction of asthma attack frequency (78). When we attempted to replicate this study in pediatric asthma (79), we actually found that sputum cellular phenotypes were very variable in the same individual over time, in both severe and mild-moderate asthma (80). These discrepancies are unexplained, but underscore the importance of not critically extrapolating from adults to children. Likely sputum cell counts reflect not merely the underlying disease, but environmental factors and treatment adherence. The U-BIOPRED group used breath-omics (the eNose) to define three clusters, but also noted that many patients changed cluster as peripheral blood eosinophil count changed over time (81). Cluster stability is discussed in more detail below.

#### Fluctuation Analyses

The first major paper used time series analyses of fluctuations in peak flow to develop a quantitative basis for objective risk prediction of acute asthma attacks and for evaluating treatment effectiveness (82). Subsequent manuscripts from this group confirmed that by considering measurements of peak flow and spirometry in isolation, rather than as part of a series, resulted in important information being lost. These and other mathematical techniques could be used to predict the response to β-2 agonists (83) and whether ICS withdrawal was likely to be successful (84). Furthermore, the method distinguished between asthma and healthy controls, partly independent of atopy, and inflammation but related to the *17q21* locus (85). These mathematical techniques have not found their way into the routine asthma clinic, but clearly this sort of mathematical analysis is giving potentially important information about asthma pathophysiology which may aid management.

### Summary: What Is Current Best Practice?

The days of diagnosing and treating asthma without making objective measurements are past. Is there any other chronic condition in which simple tests are available, but are routinely not performed before committing a child to long term treatment? We should use our knowledge of the pathophysiology of asthma to phenotype the airway, even in young children, focusing on the treatable traits. It is out with the scope of this article, but we should also consider any extra-pulmonary co-morbidities and social and environmental factors when planning management.

## Special “Asthmas”: How Does the Lancet Commission Help us?

Most of the phenotyping issues arise in children who solely have an airway disease. However, there are some situations in which airway phenotyping is helpful in understanding pathophysiology and planning treatment.

### Pre-school Wheeze: Is It Asthma, Dr?

As discussed, this question is without meaning, as is the statement that asthma cannot be diagnosed until a given and entirely arbitrary age. The proper approach is to determine which (if any) treatable traits the infant exhibits. The INFANT study (23) was discussed above; so at the very least, rather than asking pointless questions or making fatuous statements, a blood eosinophil count should be performed and aeroallergen sensitization determined. Ideally spirometry should be measured, but at the very least, if the child becomes acutely wheezy, this should be documented by a physician, and the response of wheeze intensity and oxygen saturation to inhaled β-2 agonist determined. If airway infection and PBB is suspected, documentation with cough swab or induced sputum that there is actually infection present should be mandatory, especially if multiple or prolonged antibiotic courses are being used.

### Wheeze in the First Year of Life: What Is It, Dr?

Very little is known about this asthma. We know it is not related to airway eosinophilia (40), so ICS should not be prescribed. We know it is common, and the prevalence across the world is highly variable (86). This is an area where a lot more work is needed on pathophysiology.

### Does the Child With Another Pulmonary or a Systemic Disease Also Have Asthma?

Very frequently, ICS are prescribed to children with reasons other than asthma for being symptomatic, often either preceding the realization that a non-asthma disease is present, or on the “just in case” principle. This is to be deplored; ICS have potential serious adverse events, not least the increased risk of pneumonia (87), tuberculous (88) and non-tuberculous *Mycobacterial* infection (89), at least from adult studies.

#### “Asthma” in Other Pulmonary Diseases

The classical quandary is whether survivors of premature birth have “asthma” (90). Again, phenotyping resolves this. It is clear from a number of studies that these children have fixed and variable airflow obstruction (91, 92). However, FeNO is normal not raised, even in the absence of ICS (93), nor is there an elevation of exhaled breath temperature (94), a non-specific sign of inflammation (“calor” in the parlance of yesteryear). Thus, unless there is evidence in a given individual of a second diagnosis of atopic, allergic asthma (raised FeNO and peripheral blood eosinophil count, aeroallergen sensitization), ICS should not be prescribed. The same principles of investigation apply to other airway diseases, such as obliterative bronchiolitis and the airway disease after neuroendocrine cell hyperplasia of infancy; these are discussed in detail elsewhere (95).

#### “Asthma” in Children With a Systemic Disease

Exemplar diseases are CF, primary ciliary dyskinesia (PCD) and sickle cell anemia (SCD). We have shown that the prescription of ICS in PCD is haphazard and bears no relationship to any marker of atopic sensitization or airway eosinophilia (96). In both CF and PCD, where there is evidence that airway neutrophilia leads to tissue damage from the release of proteases and other enzymes, inhibiting neutrophil apoptosis with ICS (above) may have particularly adverse consequences. In these situations also, determining the presence or otherwise of the treatable traits airway eosinophilia and β-2 responsive bronchoconstriction should be used to determine treatment. SCD is a particularly interesting airway disease. Compared with controls, SCD children had fixed but not variable airflow obstruction, and no evidence of AHR or airway eosinophilia (97) we speculate that the airway disease may be on the basis of airway ischaemia due to microinfarcts secondary to sickling, analogous to what is seen more dramatically when the bronchial arteries are stripped off the airway during the unifocalisation procedure (98). So in all systemic conditions, if a treatable trait is present it should be treated, but treatment for a non-existent problem should be withheld.

#### Obesity Asthma—Not Lean Asthma in a Fat Body

The impact of the obesity epidemic across the world is well known, and the question as to whether obesity “causes” asthma is hotly debated (99). Again, phenotyping and looking at pathophysiology sheds light on the subject. The first question in a child who is breathless and obese is, does the child have an airway disease at all? Even in lean children, exertional breathlessness is more often due to deconditioning than asthma or exercise-induced laryngeal obstruction (EILO), and many non-asthmatics were treated with inhaled medication (12). A cardiopulmonary exercise test with measurements of any post-exercise bronchoconstriction may be informative.

If the obese child truly has an airway disease, then its nature should be characterized. Of course, obesity does not prevent the development of atopy, and the child may have standard atopic, eosinophilic pediatric asthma. However, obese asthma may be relatively ICS resistant, suggestive of another phenotype in some cases. Dysanaptic airway growth is defined as a normal FEV_1_ with a greater than normal FVC, and thus a reduced FEV_1_/FVC ratio (100). Essentially airways are of normal caliber but increased length, the latter thought to be determined by lung size. In a study of six adult cohorts, four with longitudinal data, dysanapsis was found to be commoner in the obese, and associated with worse outcomes including severe asthma attacks and use of oral prednisolone. Studies on whether obese asthma is associated with Type 2 inflammation are conflicting (101, 102). It is well known that obesity is a pro-inflammatory condition. There are intriguing data suggesting that the airway may be the target of systemic inflammation, instead of the source of inflammatory cytokines spilling into the systemic circulation. In a study of two adult cohorts, plasma IL-6 was measured as a marker of systemic inflammation and related to BMI and asthma outcomes (103) Patients who were IL-6 high were more likely (but not inevitably) obese, and had worse FEV_1_ and more likely a history of asthma attacks. There was no relationship between IL-6 and serum IgE or sputum eosinophils, demonstrating that the effects of systemic inflammation were not mediated via Type 2 inflammation. Again these studies indicate the need to go back to pathophysiology, and the utility of airway phenotyping when considering airway disease, especially if it is non-responsive to conventional therapy.

## The Pathophysiology of Asthma Risk

Increasingly the importance of future risk as a domain to be considered in asthma management^1^. If risk is to be managed, it must be measured, and the underlying pathophysiology of the risk be understood.

### Asthma Attacks

Asthma attacks are all too common, may cause death, impair quality of life, incur a huge burden of health care cost and are associated with worsening respiratory and lung growth trajectories. Asthma attacks are not “exacerbations,” a futile word implying a reversible inconvenience (104, 105); they are lung attacks. A recent meta-analysis of the risk factors for an asthma lung attack (106), as did the UK National Review of Asthma Deaths^2^, highlighted that having had one bad attack, the patient was at high risk of having another. Many asthma fatalities related to social factors, such as poor adherence and failure to engage with regular follow up reviews. However, the underlying pathophysiology of asthma attacks is also important. Specifically, the concept that asthma control may be good, but risk of a future attack high, is pivotal.

Asthma attacks may be driven purely by respiratory viral infection, with no background Type 2 inflammation, usually in pre-school children with episodic viral wheeze (107). A huge surge in environmental allergen burden in the absence of viral infection may also rarely cause acute asthma attacks, as in the Barcelona soya bean epidemics (108), and thunderstorm asthma (109). The vast majority of attacks are respiratory viral driven in patients who have background ongoing type 2 inflammation; thus the combination of respiratory viral infection, allergic sensitization and allergen exposure was very strongly predictive of an asthma attack (110). It has been shown that using FeNO and (in adults) induced sputum eosinophil count to titrate ICS treatment leads to a reduction in asthma attacks (24). Inadequate ICS treatment (usually related to non-adherence) was another strong predictor of an asthma attack (106). Omalizumab therapy in the summer given to children on step 5 therapy who had had an asthma attack in the previous year ameliorated the autumnal rise in asthma attacks driven by returning to school and winter viral infections (111). Finally, in a proof of concept, double blind, randomized controlled study, mite impermeable bedding led to a reduction in oral corticosteroid use in the year after an asthma attack in children sensitized to house dust mite (112). This allows a risk prediction index for asthma attacks (112). The Seasonal Asthma Exacerbation Prediction Index (SAEPI) has been validated as a means of predicting children at risk for an asthma attack (113, 114). For those aeroallergen sensitized, an attack in the prior season and reduced spirometry predict a further asthma attack irrespective of season. Measures such as increased numbers of positive allergen skin prick tests, high prescribed ICS doses, increased FeNO, blood eosinophil counts and total and specific IgE levels may predict a seasonal asthma exacerbation. In summary, uncontrolled Type 2 inflammation, even in the face of good asthma symptom control, is a major risk factor for future asthma attacks.

There are other factors of importance which have been reviewed elsewhere (115). There are some asthma patients who never have an attack, implying either genetic protection or susceptibility factors, which are poorly understood. One example is the gene for the epithelial protein CDHR3, which is the receptor for RV-C (116), and gene mutations may convey increased susceptibility to attacks (117, 118). Indoor and outdoor air pollution, including tobacco, and vitamin D deficiency potentially through multiple immunological and other pathways (119), are all associated with increased risk of asthma attacks.

Interestingly, many severe asthma patients never have an attack, for reasons which are unclear. Analysis of the SARP-3 cohort showed that nearly half never had an asthma attack, but a quarter had at least three attacks per year (120). Peripheral blood eosinophil count, body mass index, and bronchodilator responsiveness were positively associated with frequency of attacks, but not asthma duration, age, sex, race, and socioeconomic status. The findings were replicated in previous SARP patient cohorts.

### Adverse Trajectories of Lung Function

There is an extensive literature on tracking of lung function, from a series of overlapping birth cohorts (121). Although there are discrepancies, some due to methodological issues such as lung function measurements in infancy, the balance of the evidence is that spirometry tracks from the pre-school years to late middle age at least, with possible deviations from tracking if puberty is late with a subsequent fast growth trajectory (122). In summary, spirometry rises to a plateau at about 20–25 years of age and thereafter declines (123). Adult studies have shown that failure to reach a normal spirometric plateau carries a 26% risk of COPD, compared with a 6% risk in those who attain their full growth potential (and who develop COPD because of an abnormally rapid rate of decline of spirometry) (124). A number of cohort studies have shown that some children have persistently low spirometry during childhood, putting them in the high risk category. The pathophysiology of this phenotype is poorly understood (125–127). The Tucson group used latent class analysis to determine that there were two trajectories, normal, and low lung function. Risk factors for the low lung function group included a history of maternal asthma (20.0 vs. 9.9%; *P* = 0.02); early life RSV lower respiratory tract infection (41.2 vs. 21.4%; *P* = 0.001); and physician-diagnosed active asthma (whatever the value of that label) at age 32 years (43.9 vs. 16.2%; *P* < 0.001) (125). In the Tasmanian cohort (126), there were three low trajectories (early below average, accelerated decline; persistently low; and below average); predictors included childhood asthma, bronchitis, pneumonia, allergic rhinitis, eczema, parental asthma, and maternal smoking. This group were followed up into the sixth decade, and COPD risk could be calculated. Odds ratios were 35·0, 95% CI 19·5-64·0 (early below average, accelerated decline): 9·5, 4·5–20·6 (persistently low); and 3·7, 1·9–6·9 (below average). In a combined analysis of the MAAS and ALSPAC cohorts (127), the persistently low trajectory was associated with severe wheeze attacks, early allergic sensitization, and tobacco smoke exposure. However, although it is clear that there is a group of asthmatics with low trajectory lung function who are at risk of COPD, it is not clear that anything can be done to reverse this. The Tasmanian group showed that risks were exacerbated in those children who went on to smoke, and certainly general advice should be given about risk avoidance; but this is clearly an area of asthma pathophysiology which merits further work. Also of note, and meriting further work is the association of high early all-cause mortality in populations with impaired spirometry (128, 129); it may well be that low spirometry should be used as an important signal of systemic disease (130).

### Risk of Progressive Disease

Many if not all children who develop atopic eosinophilic asthma by school age start with acute discrete episodes of pre-school wheeze before progressing to a multiple trigger pattern of symptoms. A proportion of children with viral wheeze progress to school age eosinophilic airway disease. The pathways to progression are very poorly understood (40, 107, 131, 132). Important factors associated with progression include multiple early atopic sensitization and severe attacks of wheeze (133). We know that those with no personal or family atopic history are unlikely to progress, but although predictive indices (134–136) have a good negative predictive value, unfortunately the positive predictive value not much better than 50%. Currently we know that in the pre-school years those who develop school age asthma lose lung function, which is never regained throughout life (137, 138); and they develop airway remodeling and eosinophilic inflammation. Although there is active research in the field (139) we do not have good predictive biomarkers nor do we understand the endotypes of progression or regression of the disease, nor do we have any therapeutic interventions. We know that ICS used early are not disease-modifying (140–142), but we do not know what might be useful. There are tantalizing hints that risk reduction is possible, from a randomized controlled trial of fish-oil supplementation (143) and the differences in atopic risk between the Amish and Hutterite communities, related to environmental exposures (144). This is another aspect of pathophysiology that requires more work.

### Risk of Side-Effects

Clearly ICS doses should be the minimum required to control Type 2 inflammation. There is some evidence that the risk of side-effects relates more to an excessive dose of ICS relative to the degree of airway inflammation rather than the absolute dose prescribed. In a group of adult asthmatics, there was no difference in the pharmacokinetics of an intravenous dose of fluticasone, with similar area under the curves (145). However, when the same dose was given by inhalation, there was far greater absorption into the systemic circulations in the non-asthmatics. There was unfortunately no objective measurement of inflammation, but it is not unreasonable to suppose that overdosing relative to inflammation leads to side-effects.

## Conclusion: Airway Phenotyping

Clearly we can learn a lot about the pathophysiology of the asthmas, and use this knowledge to improve diagnosis and treatment. We are still not achieving this in routine clinical practice, and this is shameful. However, even if we were phenotyping all patients and understanding pathophysiology with currently available tools, we need to progress to the next step, namely endotyping. The current position is described in the next section of this article.

## Our Target: Endotyping the Airway

The general approach to airway endotyping has been to collect and characterize as far as possible large groups of patients, for example the U-BIOPRED (146, 147) and SARP (148) cohorts, and use sophisticated—omics technologies to perform cluster analyses to try to determine the endotypes driving disease. However, caution is needed; whether a child is in a particular cluster will be driven by the underlying endotype, but also the effects of adverse environmental, infective or other factors which may vary over time, the contrasting effects of prescribed treatment, and whether the treatment is actually used by the patient. Environmental factors are unstable over time, and changes may be dramatic (108, 109, 149, 150). Treatment adherence is highly variable, difficult to measure and will affect the airway. Hence an eosinophilic airway may be the final common pathway of combinations of a steroid resistant endotype; poor adherence to ICS treatment; and increased environmental allergen exposure. Clearly the management of these three factors is very different. Very few groups have studied longitudinal stability of phenotypes or endotypes, and a second, validation cohort is rarely used; when done, the evidence for stability is weak (151). Thus, our challenge is to differentiate what truly reflects a real endotype and what represents non-disease attributes (above). This is rarely addressed or even often identified as a problem.

Furthermore, most of these analyses are in patients with severe asthma, because mild asthma hardly merits study. However, most patients referred with “severe” asthma would have mild asthma if they took their treatment (152–154). In this regard, in a recent GWAS there was substantial overlap between mild and moderate to severe asthma (155). Although the authors speculated that this related to epigenetic silencing of genes, and was therefore not reflective of gene expression, an alternative explanation is that many diagnosed as moderate to severe asthma in fact were non-adherent to treatment.

### Endoyping Asthma With Omics Technology

The U-BIOPRED (146, 147) and SARP (148) investigators, who have largely focused on severe asthma, albeit with additional controls, are the major groups exploiting -omics technology. Systems biology is increasingly used to allow clusters and phenotypes to emerge from the data (103, 156) using an unbiased analysis, not confounded by pre-set ideas. This is impossible; there is inevitable investigator bias in selecting the data to collect and analyse. For example, until relatively recently, bacterial infection was not thought relevant to asthma, but this has been challenged (above); prior to this work, bacterial samples would not have been collected and analyzed.

### Cluster Analyses: What Do They Tell Us?

The SARP investigators (157) identified four clusters: mild to moderate early onset asthma, normal body mass index and no or eosinophilic airway inflammation; the second had the same inflammatory characteristics, but the patients older, more likely to be obese, with impaired airway obstruction on spirometry and African Americans were over-represented. The last two exhibited had predominantly neutrophilic sputum; sputum eosinophilia was also sometimes seen. One of these clusters also contained older patients who were more likely to be obese and have severe asthma, obstructive spirometry and to be treated with oral corticosteroids. The final cluster was the oldest, with males over-represented, and more likely to be obese and prescribed complex medication regimes. As well as a lack of replication and assessment of cluster stability over time, the investigators could not dissociate the effects of disease from those of treatment. Furthermore, association does not prove causation, which could not be determined. Critically, this sort of cluster analysis did not appear to help us make progress in understanding or treating disease. This last point is underscored by another SARP analysis revealing this time five clusters (120) with no differences in outcomes, again questioning the usefulness of this approach.

The U-BIOPRED investigators identified four clusters in adults using sputum cell transcriptomics (158, 159). They used clinical clustering and training and validation cohorts to define phenotypes (159) which were then used to assess differences in sputum proteomics and transcriptomics data. The first were well-controlled patients with moderate-to-severe asthma. The second was in smokers with late onset severe asthma, further characterized by chronic airflow obstruction. The only difference between this and the third cluster was that the latter contained non-smokers. The fourth cluster contained obese women who had uncontrolled severe asthma, normal lung function but multiple asthma attacks. There were differences in gene expression in these clusters, which adds validity to their findings.

Assessment of the temporal stability of clusters was performed by the ADEPT group (160). They performed a baseline cluster analysis in adults which were then re-assessed over time and also validated in a U-BIOPRED subset (161). They used sophisticated mathematical techniques which included fuzzy-partition-around-medoid clustering. They included including clinical and biomarker profiles. They also classified the patients into TH2_hi_ and TH2_lo_ using gene expression profiles on bronchial biopsies. They identified four phenotypic clusters. The first was characterized by mild, early onset disease, good spirometry, and little in the way of inflammation; that present was predominantly Type 2. The second contained moderately well controlled asthmatics who had mild airflow limitation and moderate airway responsiveness; they also had Type 2 inflammation. The third group were only moderately well controlled, had minor Type 2 inflammation or a non-eosinophilic, neutrophilic phenotype airway phenotype with predominantly fixed airflow obstruction. The fourth cohort had severe asthma with uncontrolled reversible airflow obstruction, a mixed and type 2 inflammatory picture. However, there was huge overlap between the clusters for almost every marker; this does not invalidate the study, but suggests that cluster analysis may be excellent for determining groups linked by common mechanisms, but has yet to been shown in any cluster analysis. Hence the role of cluster analyses is yet to be defined. There are commonalities and differences between studies, and they have not delivered endotypes.

### Asthma Diagnosis Using Omics Technology

It is well known that asthma is poorly diagnosed (162–164), often objective diagnostic tests are not used, and those that are available are so crude when compared with the gene signature approaches used, for example, in tuberculosis diagnostics (165, 166). Blood transcriptomics would be the ideal. Red cedarwood triggered asthma in adults has a gold standard diagnostic test, unlike most of the asthmas outside the workplace, namely bronchial challenge. In a small study split into two cohorts, discovery and validation, adults with red cedarwood asthma could be reliably diagnosed using a gene signature in peripheral blood (167). Confirmation in other settings is needed, but a gene signature approach would be a major step change on current diagnostic approaches.

The U-BIOPRED group (168) also used a training and validation set. They identified 1693 genes differentially expressed in adult asthmatics as against controls, with a bigger effect size in severe asthmatics. Unfortunately, and reducing the value of this approach, around 90% of the differences could be related solely to differences in peripheral blood white cell count. Pathway analysis showed that genes related to chemotaxis, migration and myeloid cell trafficking, and decreased development of B-cells, haematopoietic progenitor cells and lymphoid organs were involved in the differences, in both training and validation cohorts. The results were similar but less pronounced in mild-moderate asthmatics. Gene signatures of corticosteroid responsiveness also differed. However, the transcriptomics did not map to any clinical cluster (169). Again this calls into question the utility of this approach, certainly as a clinical tool, and highlights our lack of understanding of the complexity of asthma. It is possible that the results might have been different if airway cells had been used, as being closer to the pathological process.

### Asthma Pathophysiology: Hypothesis Generating Studies

Gene expression is regulated in part by non-coding RNA, and this has been a subject of asthma research. In adults with severe asthma, activation status of CD4 and CD8 lymphocytes was related to non-coding RNA expression. There were significant changes in CD8 but not CD4 cells, Multiple pathways involved in T-cell activation were enhanced and there were many changes in miRNA expression (170). This is observational study, and very preliminary, but an important starting point. The rapidly expanding field of the role of micro-RNAs has been reviewed in detail elsewhere (171).

### Asthma Pathophysiology: Exploring Endotypes of Inflammation

Although the ideal is one endotype susceptible to a single biological, the reality is likely to be much more complex. Cytokines and chemokines were measured in sputum from subjects in the SARP group with varying severities of asthma, and unbiased factor analysis was used to try to define specific inflammatory pathways (172). There were complex inflammatory protein interactions identified by factor analysis. Severe asthma patients had nine increased and four decreased proteins compared to mild asthma subjects. Twenty-six mediators were significantly associated with an increasing single induced sputum leucocyte type: sixteen with neutrophils; 5 with lymphocytes; IL-15 and CCL15/MIP1δ with macrophages; interestingly, only IL-5 with eosinophils; and IL-4 and TNFSF10/TRAIL with airway epithelial cells. Forty three cytokines, chemokines, and growth factors which had no missing data were mapped onto the first 10 factors, containing mixes of Th1, Th2, Th9, and Th17 inflammatory and anti-inflammatory proteins, rather than pure pathways. Hence focus on a single specific mediator or pathway is likely an oversimplification of the complex reality of the asthmatic airway.

In a further study, the U-BIOPRED investigators started by defining phenotypes from sputum cytology, either eosinophil- and neutrophil-predominant. Next, they used sputum plugs to generate Affymetrix arrays and analyzed the data were analyzed using hierarchical, unsupervised clustering. They identified three transcript associated clusters (TACs). The first was contained oral corticosteroid dependent patients who had frequent asthma attacks, severe airflow obstruction, and the highest sputum eosinophil counts and FeNO levels. Immunologically, the receptors Il33R, CCR3 and TSLPR were upregulated and there was the strongest IL-13/TH2 and ILC2 gene signatures. The second cluster was clinically characterized by sputum neutrophilia, a raised serum CRP and eczema, and immunologically by IFN-, TNF-α- and inflammasome related genes being upregulated. The final cluster had moderate sputum eosinophilia and better spirometry, but despite sputum eosinophilia was immunologically characterized by upregulation of genes of metabolic pathways, ubiquitination and mitochondrial, with surprisingly, no TH2 signature. This important paper again highlights that eosinophilia is not synonymous with TH2 activation, confirming our own findings in severe asthma (26).

A pioneering study used transcriptomics of bronchial brushings and biopsies to determine TH2_hi_ and TH2_lo_ subgroups of mild to moderate asthmatics based on TH2 gene signatures (173). The TH2_hi_ subgroup had elevated peripheral blood levels of periostin (which is also derived from growing bone, so cannot be used in pediatrics), CLCA1 and Serpin B2, and eosinophilic airway inflammation which was ICS responsive. A subsequent study (174) using airway epithelial cell gene expression in adults confirmed this finding, but found that non-invasive biomarkers such as periostin were not sufficiently sensitive. The U-BIOPRED subsequently identified two steroid-resistant, eosinophilic subgroups in severe asthmatics (175); one with high mucosal eosinophilia, raised FeNO, asthma attacks and oral corticosteroid use; by contrast, the second eosinophilic group was more obese. We previously noted that sputum and BAL eosinophils correlate with each other, but not with mucosal biopsy eosinophils (15), but which is most important under what circumstances has not been determined. The U-BIOPRED investigators also described two non-eosinophilic groups, and developed model to predict the likelihood of the patient being steroid responsive. It is very clear that there are non-inflammatory phenotypes of severe asthma, and also that mucosal and BAL eosinophilia is not synonymous with Type 2 inflammation (176).

A further U-BIOPRED study highlighted the IL6 pathway as a potential cause of eosinophilic inflammation independent of TH2 cytokines (177). Activation of IL-6 trans signaling in air-liquid interface cultures of bronchial epithelial cells reduced the integrity of the epithelium. Associated with this was a specific signature enriched in airway remodeling genes. This signature identified a subgroup of adult asthmatics with increased epithelial expression of these inducible genes in the absence of systemic inflammation. There was an overrepresentation of patients with frequent attacks, peripheral blood eosinophilia, and submucosal of T cells and macrophage infiltration. TLR receptor pathway genes were upregulated, but cell junction genes expression was reduced. Sputum sIL6R and IL6 levels correlated with sputum markers of innate immune activation and airway remodeling. This study further evidence that there is a subset of asthmatic patients with no evidence of Type 2 inflammation; it may be that IL6 is driving airway inflammation and epithelial dysfunction in this group of patients.

The IL1 pathway may also be important (178). Sputum transcriptomics were compared in severe and mild-moderate adult asthmatics with eosinophilic and neutrophilic asthma. The investigators reported that IL1RL1 gene expression was associated with severe eosinophilic asthma, whereas NLRP3 inflammasome expression was highest in those with severe, neutrophilic asthma. These changes were only seen in induced sputum, not in bronchial brushings or biopsy specimens, underscoring the need to study multiple tissues if pathophysiology is to be understood.

Finally, FeNO is a well-known as an asthma biomarker, but whether more than one pathway results in increases has been little studied. The SARP team used a microarray platform to relate FeNO to bronchial airway epithelial cell gene expression (179). They identified 549 genes whose expression correlated with FeNO. They used k-means to cluster the patient samples and found that a total of 1,384 genes were identified in nine gene groups. Although type 2 inflammation genes were present, novel pathways, including those related to neuronal function, WNT pathways, and actin cytoskeleton, were also discovered, suggesting novel and as yet poorly characterized inflammatory pathways were at play in asthma.

Taken together, these studies suggest that in particular in severe asthma, there are multiple endotypes, possibly co-existing in some patients. There is far more to asthma pathophysiology than Type 2 inflammation. We have much more to learn from harnessing omics technology to the study of the asthmas.

### Asthma Pathophysiology: Persistent Airflow Limitation

As discussed above, there are multiple contributory factors to persistent airflow limitation, including congenital and acquired remodeling, so it is likely that multiple genes are involved. The UBIOPRED group (180) used Gene Set Variation Analysis (GSVA), as a means of detecting underlying endotypes in such heterogeneous samples. Severe adult asthma patients from the U-BIOPRED cohort with persistent airflow limitation defined as post-bronchodilator FEV_1_/FVC below the lower limit of normal) were compared with asthmatics with normal spirometry. Gene expression was assessed on the total RNA of sputum cells, nasal brushings, and endobronchial brushings and biopsies. Fourteen differentially enriched gene signatures were identified that were associated with ICS, eosinophils, IL13, IFN-α, specific CD4^+^ T-cells and airway remodeling. There was a differentially expressed gene network associated with remodeling solely in the airway wall.

### Asthma Attacks: Can We Do Better?

Chitinase-like protein YKL-40 modulates airway inflammation and serum levels are associated with asthma severity (181). In another SARP study (182), adult asthmatics were analyzed to determine if there were clusters based on YKL-40 levels, and the findings were validated in SARP. Sputum transcriptome analysis were used to demonstrate molecular pathways associated with YKL-40 clusters, of which four could be identified. Those with high serum YKL-40 were associated with earlier onset and longer duration of disease, severe airflow obstruction, and near-fatal asthma attacks. The cluster with the highest serum YKL-40 levels had adult onset disease and less airflow obstruction, but frequent attacks. Interestingly, and despite the fact that attack frequency was an important correlate of these clusters, an airway transcriptome analysis showed activation of non-type 2 inflammatory pathways. This study provides further evidence for the importance of non-TH2 pathways, and, although this needs validation, possibly suggests that serum YKL-40 levels may help risk-stratify patients.

### Future Risks: Progression From Pre-school Episodic Wheeze to School Age Eosinophilic Airway Disease

This is an extremely complicated subject which is largely beyond the scope of this review. We know that antenatal and postnatal tobacco and pollution exposure are important factors impacting future lung health, but we know little or nothing of the molecular pathways to disease [see review Bush (121)]. Furthermore, although we can predict who are low risk children, we are poor at predicting high risk, what the pathways to eosinophilic asthma actually are, and how we can reduce risk, either on a population or individual level. We have some largely descriptive –omics data which hint at pathways, but our knowledge gaps are huge.

Gene expression profiles were studied in transient and persistent wheezers using peripheral CD4+ve cells, and compared to normal, non-wheezing controls (183). The study was observational and descriptive, but did describe differences in gene expression between the two wheezing groups, with some commonalities in the paths involving proliferation and apoptosis of T-cells. Another group prospectively followed 202 preschool wheezers to school age, and testing the hypothesis that the use of volatile organic compounds (VOCs) and exhaled breath condensate would enhance the prognostic value of conventional predictive indices (184). They showed that VOCs and possibly inflammation related genes (TLR-4, catalase, TNF-α) improved predictive of persistent wheeze, but this study is also realm, and hypothesis generating, requiring validation in another cohort.

## How Will We Make OMICS Work in Practice?

When clinically indicated, invasive techniques can be used to discover novel mechanisms and pathways, but these will be only applicable in really severe cases, not in more mildly affected infants and children. For most cases, non-invasive approaches must be found, especially in children. Blood, urine and induced sputum can and should be routine clinical tests, there are other accessible biosamples which should be evaluated. Exhaled breath analysis is non-invasive, requires only passive co-operation, and with modern analytical techniques can give point of care answers. Investigators can distinguish different airway diseases in adults (COPD, asthma) from a breathprint of VOCs (185, 186). In children, 8 of 945 compounds studied could differentiate asthmatics from controls with a sensitivity of 89% and a specificity of 95% (187). There has been considerable interest in sophisticated mass spectrometry techniques, which can be applied for example to skin secretions, in order to detect airway infection in CF (188). The true test of the utility of these techniques will be whether they can differentiate children with non-specific respiratory symptoms from true asthmatics, and predict steroid responsiveness in the asthmatics. Perhaps in the future we will have a pediatric “Breathalyzer” which will give a readout of the important biomarkers to tell us the diagnosis, what endotypes are at play, and how best to treat the child.

## Summary and Conclusions

There is no doubt we have incredible opportunities within reach to transform the diagnosis and treatment of the asthmas. We have powerful tools and –omics technologies available to us, as well as pathway specific monoclonals. These need to be targeted rationally. We need to reflect that the specific designer molecules which are transforming CF (2–4) would have been discarded as ineffective if they had been applied indiscriminately to all CF patients, and not to gene class specific sub-endotypes. The CF community are progressing to *ex vivo* testing of novel compounds (189, 190) and we must do the same for the asthmas, to produce truly personalized airway medicine.

There are questions specifically pertaining to the asthmas that we need to address. We need to understand steroid resistant, particularly non-TH2 driven eosinophilia, and apparently non-inflammatory asthma pathways. We have argued elsewhere that children with refractory difficult asthma (for example, those persistently not taking basic medications) should not be denied biologicals to prevent them from dying (191); even in this group, identification of the endotype will be needed to ensure the right child gets the right biological. But we also need to appreciate the diversity of the asthmas—Type 2 inflammation, although obviously important, is only one part of the picture, and we need to better are specific appreciate the whole.

But finally we must appreciate that the more sophisticated and expensive approaches to monitoring and treatment are available, the more clinical skills become relevant (192). We will need better get the basics right, rather than immediately deploy the latest gene probe test and expensive therapeutic molecule. At bottom, most pediatric asthma is a simple disease to diagnose and treat if basic measurements are made and the child is given low dose therapy appropriately and regularly. We should never lose sight of this reality, and never stop using our clinical skills, and honing those skills, to get the basics right, working in a multidisciplinary team alongside the child and family.

## Author Contributions

The author confirms being the sole contributor of this work and has approved it for publication.

### Conflict of Interest Statement

The author declares that the research was conducted in the absence of any commercial or financial relationships that could be construed as a potential conflict of interest.

## Footnotes

^1^https://ginasthma.org/

^2^https://www.rcplondon.ac.uk/projects/national-review-asthma-deaths/
